# The dual regulatory role of miR-204 in cancer

**DOI:** 10.1007/s13277-016-5144-5

**Published:** 2016-07-20

**Authors:** Tianqi Li, Hongjie Pan, Runsheng Li

**Affiliations:** Key Laboratory of Reproduction Regulation of NPFPC, SIPPR, IRD, Fudan University, Shanghai, China

**Keywords:** miR-204, Target gene, Cancer, Tumor-suppresive gene

## Abstract

MicroRNAs (miRNAs) are a group of endogenous, small (about 22 nucleotides) non-coding RNAs which negatively regulate gene expressions. As one of them, miR-204 originates from the sixth intron of the transient receptor potential melastatin 3 (*TRPM3*) gene. Therefore, expression of *miR-204* is under the control of the *TRPM3* promoter and regulated by genetic and epigenetic mechanisms. miR-204 has been found to play the important roles in development of eyes and adipogenesis. Its pathological functions have been observed in a few diseases including pulmonary arterial hypertension, diabetes, and various types of cancers. It is believed that miR-204 acts as a tumor-suppressor via promoting apoptosis, conferring the resistance of cancer cells to chemotherapy, and suppressing the self-renewal of cancer stem cells (CSCs) and the epithelial to mesenchymal transition (EMT). Expression of miR-204 is repressed by its targets XRN1 and TRKB in prostate cancer and endometrial carcinoma, respectively; therefore, they establish an oncogenic feedback loops that play an important role promoting development of cancer. In this review, we summarize our current knowledge regarding miR-204, including its expression, regulation and biological functions, especially focusing our discussion on its role in tumor development and tumor progression.

MicroRNAs (miRNAs) are a non-protein coding class of small regulatory RNAs (22-nucleotides long) that play an essential role in post-transcriptional regulation of gene expression through binding to the 3′-untranslated region (3´-UTR) of messenger RNAs (mRNAs). The mechanism of mRNA silencing depends on the degree of complementarity of the seed sequence to the 3′-UTR of the target mRNA [1]. When perfect base-paring homology exists between a miRNA and a mRNA, the RNA-mediated interference pathway is induced, which eventually results in cleavage of mRNA. When binding is imperfect, which happens more commonly, the translation of target mRNA is regulated without significantly affecting the corresponding level of mRNA. On average, each miRNA regulates approximately 200 target mRNAs and ~10–40 % of the mRNA sequences are roughly estimated to be targeted by microRNAs in human [2]. To date, over 2500 potential human miRNAs have been recorded in miRBase v20 and the number is still accumulating. miRNAs have been found to play integral roles in regulating an array of fundamental cellular processes, including the cell cycle, differentiation, and proliferation, thereby adjusting numerous pathways related to development and diseases.

To date, miR-204’s involvement has been demonstrated for development of the retina and eye, diabetes, many types of cancers, and other pathological processes. The present article reviews our current knowledge of the physiopathology of miR-204 with a focus on its function in different types of cancers, particularly, in prostate cancer.

## Physiological functions of miR-204

### The role in development of eyes

Genes are known to have important functions in the tissues where they are highly expressed. miR-204 is orders of magnitude more highly expressed in human retinal pigment epithelium (RPE) than in most of the 20 adult human tissues tested [3] and highly expressed in eye of zebrafish and mouse. miR-204 is one of miRNAs whose expression is regulated by different levels of light in the mouse retina. In medaka fish, Conte et al. reported that morpholino-mediated ablation of *miR-204* expression resulted in an abnormal phenotype of eye which is associated with optic fissure coloboma [4]. Recently, by studying Dicer1-deficient RPE of mice, Ohana et al. reported a key role of miR-204 in regulation of the RPE differentiation program. These reports, therefore, highlight miR-204 as a “master regulator” of the molecular networks that regulates lens morphogenesis in vertebrates.

miR-204 acts as the master regulator in the model of medaka fish, and the transcription factor MEIS2 is one of the main targets of miR-204 functions [4]. MEIS2 homeoprotein directly upregulates PAX6 [5], a lineage-specific transcriptional factor that plays a key role during vertebrate lens morphogenesis. In turn, PAX6 was shown to promote expression of miR-204 both in mice and in medaka by directly binding its promoter [6]. In addition to MEIS2, other genes including SOX11, an important factor for development of eye and the central nervous system, were also shown to be the miR-204 targets in RPE. Importantly, these miR-204 targets (e.g., SOX11, etc.) are upregulated in the lens with knockout of PAX6, suggesting that PAX6 downregulates multiple genes during eye development in the way dependent on miR-204 [6]. Together, these results strongly suggest the presence of a miR-204-MEIS2-PAX6 negative feedback loop in RPE which controls the physiological function of miR-204 in eye development.

### The role in adipogenesis

Mesenchymal stem cells (MSCs) have potential to undergo multilineage differentiation into multiple connective tissue cell types, such as osteoblasts, adipocytes, and myoblasts. Differentiation of MSCs into different lineages of cells is tightly regulated, and alteration or malfunction of this regulation could result in pathological consequences. For example, bone loss is often accompanied with the increase of bone marrow adiposity in aging [7]. Moreover, it was reported that adipose tissue in bone marrow is inversely related to bone formation in osteoporosis, and patients with high bone mass phenotype show inhibition of adipogenesis [8]. These findings suggest a switch between the osteoblast and the adipocyte differentiation of the MSCs during the development of osteoporosis or a high bone mass phenotype. Interestingly, miR-204 promotes the adipogenesis and inhibits the osteogenesis of human MSCs through the direct suppression of RUNX2 [9], a key transcription factor for osteogenesis. Therefore, miR-204 is a “switch” molecule that controls the fate choice between osteogenesis and adipogenesis [9]. Consistently, miR-204 promotes the differentiation of human adipose-derived MSCs into mature adipoctyes [10]. Moreover, disheveled segment polarity protein 3 (DVL3), a key regulator of the Wnt/β-catenin signaling pathway, was identified as the target of miR-204. In addition, overexpression of miR-204 was shown to induce the downregulation of β-catenin and the canonical Wnt target gene, CCND1, in mature adipoctyes, while its knockdown led to upregulation of CCND1. Therefore, miR-204 was proposed to regulate adipogenesis by controlling DVL3 expression and subsequently inhibiting the activation of the Wnt/β-catenin signaling pathway [10].

Pathological studies have uncovered that downregulation of miR-204 contributes to enhanced proliferation and reduced apoptosis of pulmonary artery smooth muscle cells [11], which eventually resulted in pulmonary arterial hypertension (PAH). In addition, miR-204 plays a critical role in development of diabetes. For instance, beta-cell dysfunction and impaired insulin production are hallmarks of diabetes. Beta-cell thioredoxin-interacting protein (TXNIP), a cellular redox regulator upregulated in diabetes, induces the expression of miR-204. By targeting and downregulating MAFA, a known insulin transcription factor, miR-204, blocks insulin production [12]. In contrast, TXNIP deficiency protects against diabetes by preventing beta-cell apoptosis [12]. These results demonstrated a potential anti-apoptotic role of miR-204 in beta cells.

### Roles of miR-204 in cancers

To date, most of researches regarding miR-204 have been focused on its roles in cancers. Studies have demonstrated that miR-204 has a dual function as a tumor-suppressive gene and/or an oncomiR in different cancers.

### Aberrant expression of miR-204 in cancers

The first study regarding miR-204 in cancers was reported by Rolda et al. in 2007, who observed that miR-204 is dramatically upregulated in insulinomas and that its expression level correlates with immunohistochemical expression of insulin [13]. Subsequently, it was shown that miR-204 is one of five miRNAs that most highly expressed in acute lymphocytic leukemias [14]. However, in contrast to these reports, decreased miR-204 was shown in many solid tumors, including primary melanomas glioma, non-small cell lung cancer, bladder cancer, gastric cancer, head and neck tumor, and endometrioid endometrial cancer (EEC) [15–18]. However, there are conflicting results regarding the miR-204 expression in some other solid tumors including breast cancer and prostate cancer (PCa). For example, while miR-204 expression was shown to be significantly elevated in five human breast tumors when compared to their matched adjacent non-tumor samples [19], other studies showed the opposite results indicating that not only are miR-204 expression in breast cancer specimens lower than that in their adjacent normal tissues but also the low expression of miRNA is significantly associated with a more aggressive tumor phenotype [20, 21]. Similarly, in PCa, an increased expression of miR-204 was previously observed in five tumor specimens by immunostaining [22]. In contrast to this, by analyzing a tissue microarray that included 135 PCa specimens, we showed that lower levels of miR-204 in primary prostate cancer than that in the control samples [23]. Moreover, we demonstrated an inverse correlation between and miR-204 in prostate cancer specimens and serum level of prostate-specific antigen (PSA), which is an important clinical diagnostic biomarker of PCa [23].

### Tumor-suppressive role of miR-204

Although function of miR-204 has not yet been reported in insulinomas and acute lymphocytic leukemias where it is highly expressed [13, 14], most of studies have revealed that miR-204 plays a role as a tumor-suppressive gene in all types of cancers studied. To explore its anticancer mechanism, researchers have reported 30 validated target genes of miR-204 in different cultured cells representing 19 types of cancers (Table 1). Among these target genes, the expression of BDNF [20], MEIS1 [38], FOXC1 [18], NUAK1 [40], RAB22A [42], and XRN1 [23] is inversely correlated with that of miR-204 in those tumor specimens examined, strongly suggesting that miR-204 is one of the key suppressors of the target genes. Accumulating evidence has recently demonstrated that long non-coding RNAs (lncRNAs) play a pivotal role in tumorigenesis. miR-204 was also shown to repress LncRNA HOTTIP expression via the argonaute 2-mediated RNA interference pathway in hepatocellular carcinoma [37]. This study is particularly interesting, since it provides an example to show how expression of lncRNAs is controlled by short ncRNAs, such as miR-204.

**Table 1:** Target genes of miR-204 validated in cancer cells

Target gene	Types of cancer/cells	Ref.
BCL-2	Intrahepatic cholangiocarcinoma	[24]
	Colon cancer	[25]
	Neuroblastoma	[26]
	Gastric cancer	[27]
BDNF	Breast cancer	[20]
CDC42	Nasopharayngeal carcinoma	[28]
CYCLIN D2	Retinoblastoma	[29]
EPHB2	Glioma	[30]
EZRIN	Gastric cancer	[31]
FOXC1	Endometrial cancer	[18]
FOXM1	Cholangiocarcinoma	[32]
IGFBP5	Papillary thyroid carcinoma	[33]
IL-11	Breast cancer	[34]
JAK2	Breast cancer	[35]
LC3B	Clear cell renal cell carcinoma	[36]
LncRNA HOTTIP	Hepatocellular carcinoma	[37]
MEIS1	Nephroblastomas	[38]
MCL-1	Pancreatic cancer	[39]
MMP-9	Retinoblastoma	[29]
NUAK1	Non-small cell lung cancer	[40]
PDEF	Prostate cancer	[22]
	Breast cancer	[19]
PHOX2B	Neuroblastoma	[41]
RAB22A	Colorectal cancer	[42]
	Gastric cancer	[43]
RUNX2	Prostate cancer	[44]
SIRT1	Gastric cancer	[45]
SIX1	Non-small cell lung cancer	[16]
	Breast cancer	[46]
SAM68	Breast cancer	[47]
SLUG	Intrahepatic cholangiocarcinoma	[48]
SOX4	Glioma	[30]
	Gastric cancer	[49]
	Squamous cell carcinomas	[50]
TRKB	Neuroblastoma	[40]
TRPM3	Clear cell renal cellcarcinoma	[51]
USP47	Gastric cancer	[43]
XRN1	Prostate cancer	[23]

Alterations in the apoptotic response in cancer cells can promote tumor initiation, progression, and drug resistance. Apoptosis can be triggered by different signals such as DNA damage, upon which activation of p53 protein promotes apoptosis through upregulating the transcription of several pro-apoptotic proteins in BCL-2 family members. On the other hand, by sequestering these pro-apoptotic BCL-2 family members and thereby inhibiting their pro-apoptotic activities, BCL-2 exerts its anti-apoptotic function [52]. Several studies have demonstrated that some oncogenic miRNAs are involved in the control of apoptosis and exert their anti-apoptotic effects by directly targeting pro-apoptotic BCL-2 members’ mRNAs [53]. BCL-2 has been observed to be the miR-204 target in four types of cancers in vitro (Table 1). Therefore, induction of apoptosis seems to be the common mechanism for tumor-suppressive role of miR-204. However, none of the studies thus far revealed the presence of inverse correlation between expression of BCL-2 and miR-204 [24–27]. Given that a mechanism blocking the binding of miR-204 to 3′-UTR of BCL-2 was recently proposed [54] (also shown later), a further study is needed to confirm that miR-204 is actually the key regulator of BCL-2 in vivo*.*

EMT is a unique process that describes the molecular reprogramming and phenotypic changes characterized by a transition from polarized immotile epithelial cells to motile mesenchymal cells, leading to tumor metastases. This transition is often associated with a decrease in the expression of proteins that enhance cell-cell contact (e.g., E-cadherin and *γ*-catenin, etc.), as well as with an increase in the expression of mesenchymal markers (e.g., vimentin and N-cadherin, etc). Transforming growth factor β (TGF-β) is a critical factor which induces EMT and has a key role in the bone metastatic process of breast cancer cells. In the bone microenvironment, TGF-β is released from bone during bone resorption, then it stimulates breast cancer cells to produce osteolytic factors such as interleukin 11(IL-11) to mediate osteolysis by stimulating osteoclast formation and bone resorption activity [55]. Additionally, TGF-β stimulates expression of master EMT regulators, such as SOX4 and members of the Twist, Snail families of transcription factors. Overexpression of homeoprotein SIX1, a developmentally restricted transcriptional regulator, induces EMT that is in part dependent on its ability to increase TGF-beta signaling [56]. Interestingly, IL-11 [34], SOX4 [49] and SIX1 [16] have been shown to be the targets of miR-204. Therefore, by repressing the expression of these genes, miR-204 exerts its tumor-suppressive effect via inhibiting EMT and bone metastasis of breast cancer cells [20]. Consistent with this, systemic delivery of miR-204 using a lipid-based vehicle resulted in significant reduction or elimination of lung metastases in a mouse model [20]. These results also revealed the potential of miR-204-based strategy for cancer treatment. In addition to suppressing breast cancer, miR-204 also represses EMT and metastasis of other cancer cells. For example, miR-204 inhibits invasion and EMT phenotype of esophageal cancer cells through targeting forkhead box protein M1 (*FOXM1*) gene [32], a transcription factor which plays an important role in the activation of EMT. SIRT1, a class III histone deacetylase, is upregulated in lymph node metastases of gastric cancer. By targeting SIRT1, overexpression of miR-204 decreases the levels of EMT-associated gene vimentin but increases E-cadherin levels in gastric cancer cells [45]. Consistent with this, miR-204 significantly suppresses lung metastasis of NSCLC cancer cells in a SCID mouse model [40].

A relationship between EMT and cancer stem cells (CSCs) has been proposed with evidence demonstrating that EMT cells exhibit stem cell-like traits and CSCs acquire mesenchymal-like characteristics [57]. CSCs, similar to somatic stem cells, are defined as cells within a tumor that possess the capacity to self-renew and to differentiate into the heterogeneous lineages of cancer cells that comprise the tumors [57]. CSCs have a great impact on development of tumors including tumor growth, metastasis, and recurrence due to their self-renewal potential and multidirectional differentiation ability. The 68-kDa Src-associated protein in mitosis (SAM68) is a GTPase-activating protein (GAP)-related protein involved in intracellular signal transduction that maintains the self-renewal of breast CSCs via affecting the oncogene serine/arginine-rich splicing factor. By targeting SAM68, miR-204 reduces the stem cell phenotype in breast cancer and neutralizes the tumorigenic effect of Sam68 in vivo [47]. It was also reported that miR-204 suppresses self-renewal, stem cell-associated phenotype, and migration of glioma cells via targeting the stemness-governing transcriptional factor SOX4 and the migration-promoting receptor EphB2 [30]. Together, these results suggest that miR-204 acts as a tumor-suppressive gene in CSCs.

Tumor-suppressive activity of miR-204 has been reflected by its promoting effect on chemotherapy of cancer cells as well. It was shown that overexpression of miR-204 increases responsiveness of gastric cancer cells to 5-fluorouracil and oxaliplatin treatment [27]. miR-204 also inhibits proliferation and invasion and enhances chemotherapeutic sensitivity of colorectal cancer cells by downregulating RAB22A [42]. Moreover, ectopic miR-204 expression significantly increases sensitivity of neuroblastoma cells to cisplatin and etoposide in vitro [26].

### Dual regulatory function of miR-204 in prostate cancer

Prostate cancer (PCa) is the most common malignancy affecting males in western countries, and it is the second leading cause of cancer deaths worldwide. Tremendous studies have proven that androgen, which works by binding and activating androgen receptor (AR), is critical for progression of prostate cancer [58]. Androgen deprivation treatment (ADT) has been used clinically to suppress the tumor growth and progression of androgen-sensitive prostate cancer. However, 2–3 years after treatment, many androgen-sensitive prostate cancers will eventually develop the resistance to ADT and become castration-resistant prostate cancer (CRPC), in which upregulation of androgen signaling pathway is believed to play an important role [58, 59].

**Fig. 1:**
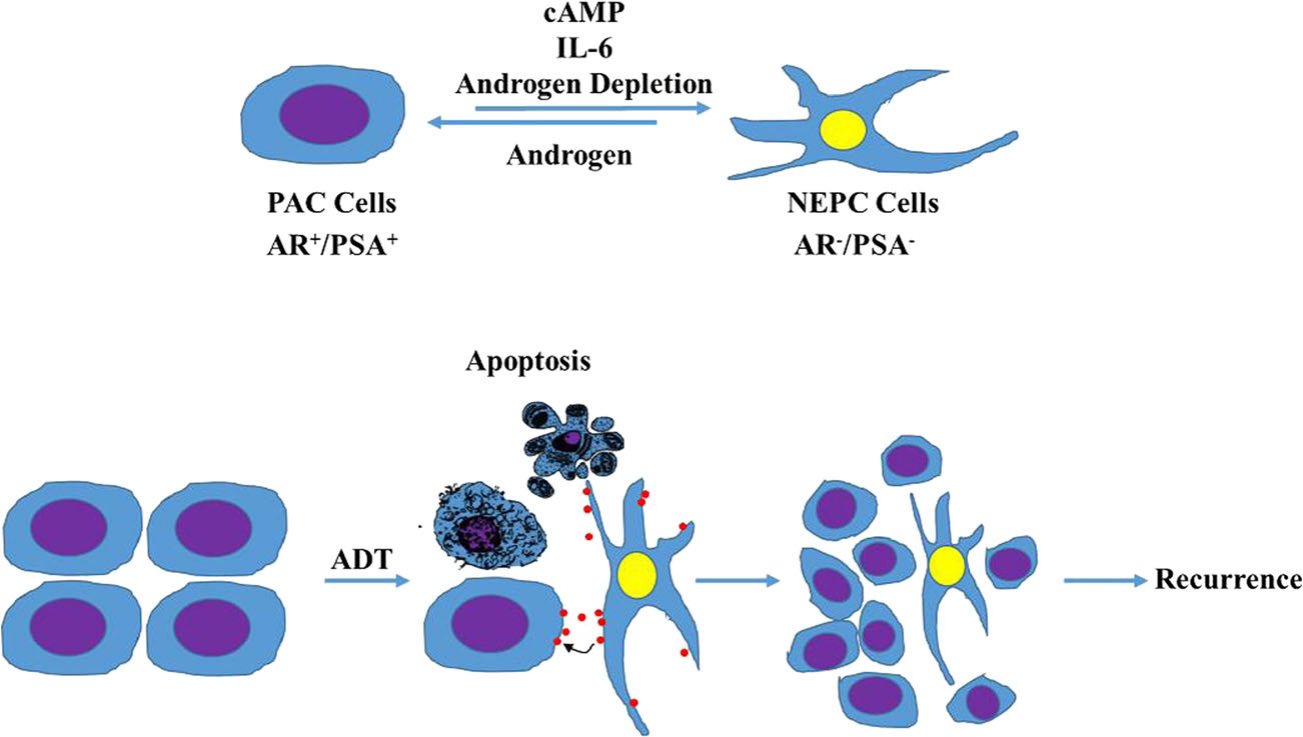
Neuroendocrine differentiation promotes recurrence of prostate cancer. The cultured PAC cells can be induced to differentiate into NEPC cells by diverse signals including cAMP, IL-6 and androgen depletion via different mechanisms. Most of PAC cells die in the patients who are treated with androgen deprivation treatment (ADT). However, ADT also causes NED. The NEPC cells secret multiple neuropeptides which promote survival and growth of PAC cells in the presence of ADT. The recurrence is eventually

Vast majority of PCa is characterized as prostatic adenocarcinoma (PAC) with luminal cell features and expression of AR and PSA [58]. Interestingly, PAC usually contains a small population (usually ~1 %) of scattered neuroendocrine (NE)-like prostate cancer (NEPC) cells that do not express AR and PSA [60] (Fig. 1). As a subtype of NEPC cells, small cell neuroendocrine carcinoma (SCNC) is often seen in patients with advanced disease and is composed of pure neuroendocrine tumor cells that express prostate CD44 [61], a marker of CSCs. NE differentiation (NED) of PAC cells to NEPC can be induced in cultured cells. For example, treatment of LNCaP cells, the most widely used PAC cell line, with cAMP (or cAMP-inducing agents) or androgen depletion induces NED within a few days [62]. Importantly, studies have shown that ADT may contribute to development of CRPC [63], in which the focal NED within the tumors increases the levels of NE-derived peptides such as neuron-specific enolase, leading to increased levels of chromogranin-A in patient serum [63]. Consistent with this, studies indicated that suppression of AR expression is required for NED of cultured PAC cells in vitro [64]. It is believed that cancerous NE cells secrete a variety of growth factors that can promote the proliferation of adjacent PAC cells through a paracrine mechanism in an androgen-ablated environment [65], accounting for androgen-independent growth of prostate cancer (Fig. 1). Therefore, it was proposed that AR can be either tumor suppressor or promoter in prostate cancer [66], depending on the pathological background.

The role of miR-204 in PCa was first reported by Turner et al., who identified that prostate-derived Ets factor (PDEF) is a target of miR-204 [22]. Given that overexpression of PDEF repressed growth of prostate cancer DU-145 cell line and PC-3 cell line, which represents the NEPC cells, and that the expression of PDEF in prostate cancer specimens is downregulated when compared with the controls [22], it suggests that miR-204 is an oncomiR through targeting PDEF and inhibiting PDEF’s tumor-suppressive function in PCa.

**Fig. 2:**
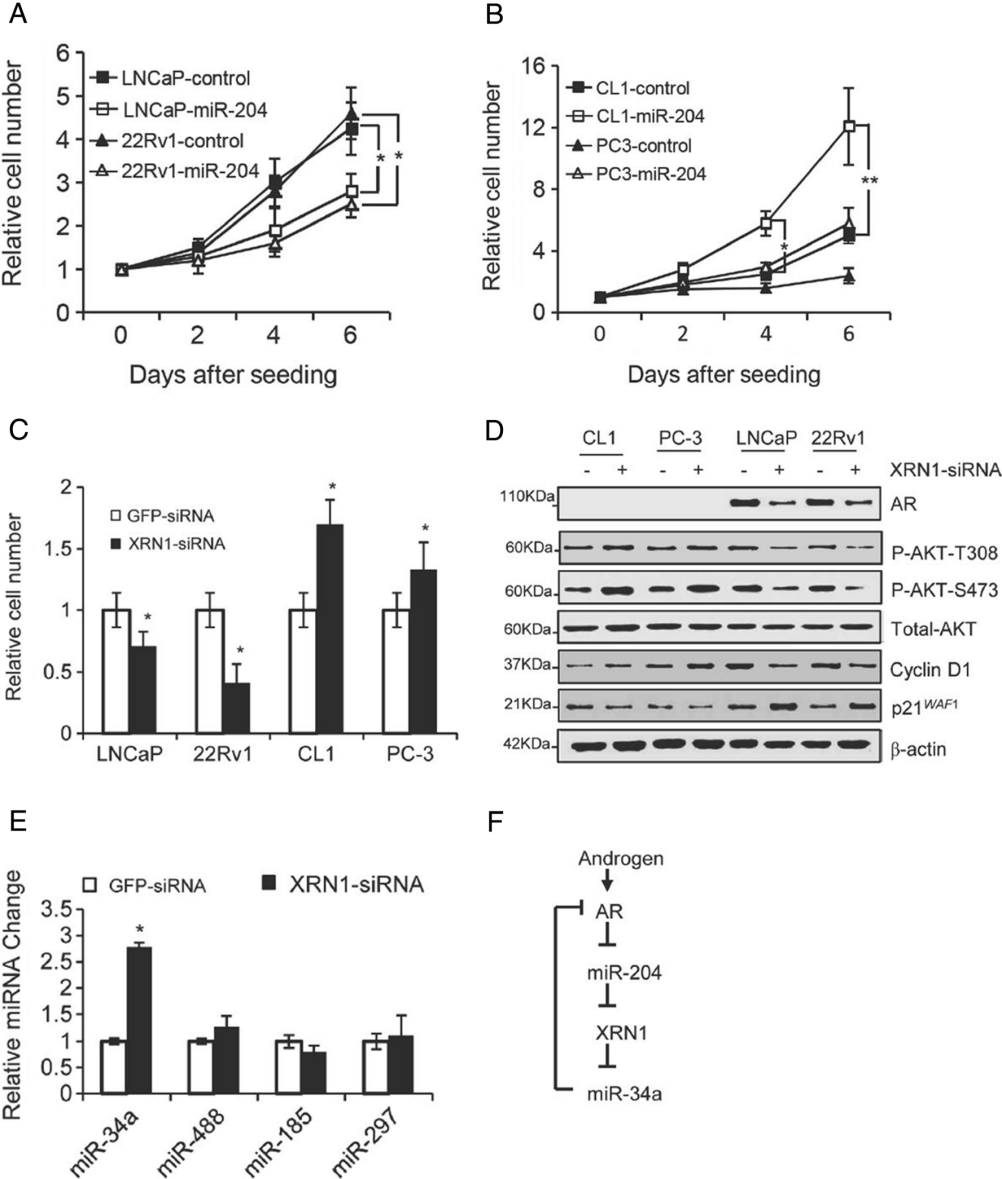
miR-204 and its target XRN1 dually regulate growth of different prostate cancer cells. **a** and **b** Cell growth of different PCa cell lines infected with the recombinant miR-204-expressing lentivirus or the control lentivirus. **c** Effect of silencing of XRN1 on cell growth. **d** Western blot analysis of prostate cancer cells transfected with XRN1 siRNA. **e** RT-qPCR assays of four AR-targeting miRNAs in LNCaP. **f** Schematic representation of the proposed AR/miR-204/XRN1/miR-34a feedback loop. The activation of the loop by androgen induces an upregulation of AR signaling. The modulation is advantageous for development of aggressive phenotype of PAC. Cited from Ding et al. [23]

By using comprehensive approaches including gain/lost of function of miR-204 and xenografts analyses, we demonstrated that miR-204 acts as an oncomiR in NEPC cells (PC-3 cells and CL1 cells) but as a tumor suppressor in PAC cells including LNCaP cells and 22RV1 cells [23] (Fig. 2). We identified that XRN1, 5′-3’exoribonuclease 1, is a miR-204 target. XRN1, as well as the other members of the highly conserved XRN1 family, degrade diverse RNA substrates during general RNA decay and function in specialized processes of RNA metabolism, such as nonsense-mediated decay, gene silencing, rRNA maturation, and transcription termination [67]. Interestingly, knockdown of XRN1 also dually regulated cell growth and colony-forming capability of NEPC and PAC cells, consistent with effects of ectopic expression of miR-204 in these PCa cell lines [23]. The dual regulatory function of miR-204/XRN1 was further demonstrated by their dually-modulating effects on expression of some key regulators of cell cycle and apoptosis, including AKT phosphorylation, Cyclin D1, and p21^*WAF1*^ (Fig. 2). In addition, we further revealed that androgen/AR inhibits the expression of miR-204 both in PAC cells and NEPC cells, subsequently leading to upregulation of XRN1 expression in a way depending on suppression of miR-204 [23]. Therefore, AR-miR-204-XRN1 axis is probably one of the key mechanisms for dual regulatory function of AR in different stages of tumor progression of PCa.

AR has also a dual regulatory function in breast cancer cells [68]. Although most studies suggested that miR-204 is a tumor suppressor in breast cancer [20, 21, 69, 46], overexpression of miR-204 was also shown to increase migration, invasion, and metastasis of breast cancer MCF-7 cells and inhibit EMT by targeting PDEF [19]. Given that miR-204 is downregulated by androgen in MCF-7 cells (our unpublished data), it will worth examining whether the above conflicting results are caused by varied AR backgrounds in different breast cells tested.

Besides XRN1, cdc42 is another miR-204 target [28] which has a dual yet opposite growth-regulatory function in cancer cells [70]. It is currently unknown whether cdc42 has a contribution to miR-204-mediated dual function in cancer cells.

## Factors regulating expression and function of miR-204

### Hereditary factor

High-resolution custom miRNA comparative genomic hybridization (CGH) of public domain datasets for several types of cancers showed that the 9q21.12 chromosomal region containing miR-204 is frequently lost in multiple types of cancers [20, 71], suggesting that loss of genomic loci containing miR-204 is directly linked with the deregulation of key tumor-suppressive pathways, leading to tumor growth and metastasis.

By binding to the 3′-UTR of target genes, miRNAs exert their expression-regulating rule. However, the interaction can be significantly affected by SNP either in miR-204 or in the 3′-UTR of targets. Recently, a new heterozygous mutation, n.37 C > T, in the seed region of miR-204 was identified in a five-generation family of an autosomal dominantly inherited condition of retinal dystrophy and bilateral coloboma [72]. Consequently, this mutation significantly alters miR-204’s targeting capabilities. In vivo injection of this mutated miR-204 in medaka fish caused a phenotype consistent with that observed in the family. This provides the first evidence of miR-204’s contribution to eye disease, likely through a gain-of-function mechanism. On the other hand, the SNPs in 3′-UTR of PHOX2B differentially affect miRNA-mediated regulation of the stability of target mRNAs [41].

### Factor blocking binding of miR-204 to 3′-UTR of its targets

Besides some SNPs, human transformer 2β (TRA2β) has been recently reported to affect interaction of miR-204 and its target by competing the binding sequence of miR-204 in a mutually exclusive way [54]. TRA2β is a serine/arginine-rich-like protein splicing factor with wide-ranging roles in gene expression as an RNA-binding protein. The consensus sequence for TRA2β-binding (GAA) lies within the miR-204-binding site of BCL-2 3′-UTR, TRA2β antagonizes the effects of miR-204 and upregulates BCL-2 expression [54]. Such a mechanism is very interesting, partially because it can explain why miR-204 regulates expression of its targets with different efficiencies in the way depending on cell lines used [44]. Given that TRA2β mRNA expression is significantly upregulated in colon cancer [54] and prostate cancer tissues compared with paired normal tissues, it is highly possible that TRA2β plays an important role in carcinogenesis by blocking function of miR-204.

### miR-204 and *TRPM3*

*TRPM3* gene encodes proteins that form cation-permeable ion channels on the plasma membrane. The *miR-204*-encoding gene is located in the sixth intron of *TRPM3.* Expression of mature miR-204 and pri-miR-204 linearly correlated with that of *TRPM3* gene both in vitro [11, 30] and in vivo [23], strongly suggesting that *miR-204* and *TRPM3* share common regulatory mechanisms. Downregulation of miR-204 in glioma [30] and NSCLC [40] was attributable to the DNA methylation of *TRPM3* promoter.

Interestingly, *TRPM3* was validated to be a target of miR-204 [51], consistent with that some genes are autoregulated via intronic microRNAs [73]. Some stimuli may induce expression of both *miR-204* and *TRPM3*. The activity of miR-204 would then contribute to downregulation of TRPM3 activity to the pre-stimulus level. Therefore, both TRPM3 and miR-204 can play their roles only temporarily in these circumstances. With the role of TRPM3 in cancers [51, 74], the TRPM3-miR-204 feedback loop may be important to prevent progression of oncogenic transformation and may also exist widely in physiological situations.

TRPM3 is overexpressed in choroid plexus papillomas, glioblastoma multiforme, and clear cell renal cellcarcinoma (ccRCC) [51, 74], suggesting that TRPM3 has a tumor-promoting role. It is currently unknown whether miR-204 expression actually increases as a result of *TRPM3* overexpression in choroid plexus papillomas and glioblastoma multiforme. ccRCC is a dominant subtype of kidney cancers in most of which the von Hippel-Lindau (*VHL*) tumor-suppressor gene is mutated or lost [75]. The tumor-suppressive function of VHL gene depends on its promoting effect on miR-204 expression [36]. Interestingly, VHL-induced expression of miR-204 correlated with expression of two short transcripts from *TRPM3*, but with not expression of the large transcript encoding the full-length protein [36]. Uncoupling of expression of TRPM3 and *miR-204* is obviously important for development of ccRCC and probably exists in other types of cancers where *TRPM3* is upregulated.

### Transcriptional repressor of *miR-204*

The signal transducer and activator of transcription (STAT)-3 plays an indispensable role in the progression of a wide variety of cancers. Activation of STAT3 is mediated by phosphorylation of latent cytoplasmic STAT3 on specific residues (Y705, Ser727) by a variety of tyrosine and serine kinases (e.g., Src and JAK2), leading to its dimerization and nuclear translocation, where STAT3 acts as a transcription factor to modulate expression of different genes including miRNA-204. The activated STAT3 binds preferentially the *TRPM3* promoter, eventually suppresses expression of miR-204 in pulmonary artery smooth muscle cells [11, 76]. Moreover, exosomes-mediated cytoprotective action of mesenchymal stromal cells induced by hypoxia-induced pulmonary hypertension depends on inhibition of STAT3-miR-204 axis [77]. Similarly, activation of STAT3 has been shown to downregulate miR-204 expression in nasopharayngeal carcinoma [28] and endometrial carcinoma [78]. Given that STAT3 is a critical mediator of differentiation, activation, migration, and inflammatory capacity of immune cells and stromal cells that create the microenvironment supporting tumor cell growth [79], it will not be surprising that miR-204 has multiple functions in cancer development.

### Positive feedback loops of miR-204 and its targets

As already observed in other miRNAs, expression of miR-204 is also regulated by its targets. The miR-204-Meis2-Pax6 negative feedback loop as mentioned above plays an important role in eye development. However, three other positive feedback loops between miR-204 and its different targets have been established. For examples, miR-204 and its target Six1 inhibit their expression mutually [46]. TrkB-STAT3-miR-204 regulatory circuitry plays an important role in promoting metastasis in endometrial carcinoma [78]. In this loop, tropomyosin-related kinase B (TRKB), a miR-204 target [40], activates the JAK2/STAT3, and further suppresses expression of miR-204, eventually resulting in an increased expression of miR-204 targets including TRKB itself [78]. TRKB, which is important for neural development and is an independent prognostic marker in many tumor types, is the receptor of brain-derived neurotrophic factor (BDNF) [80]. Given that BDNF is also a miR-204 target [20], the above loop is expanded to BNDF-TrkB-STAT3-miR-204 regulatory circuitry. However, its significance is needed to be further evaluated in different models of cancers.

Recently, we identified an AR-miR-204-XRN1-miR-34a regulatory circuitry in prostate cancer cells [23]. In this loop, AR signal represses miR-204 expression, increasing protein levels of XRN1, which then degrades miR-34a in a cell type-dependent way [23] (Fig. 2). Moreover, AR itself is a target of miR-34a as reported previously, AR signaling is therefore much enhanced in prostate cancer cells through this loop (Fig. 2). Interestingly, both of regulatory circuitries (i.e., BNDF-TrkB-STAT3-miR-204 and AR-miR-204-XRN1-miR-34a circuitries) establish the mechanism by which a weak signal generates a strong effect. Such a mechanism could make a great contribution to development and recurrence of cancers.

### Perspectives

Given the abundant literature, it is now increasingly clear that miR-204 is a tumor-suppressive gene. However, its function can also evolve towards to an oncomiR in some situations, for example, under pressure of therapy in PCa. This is a challenge for miR-204-based cancer therapies. It is expected that miR-204 also plays an important function in early development of mammalian embryos, given the reported establishment of miR-204 functions in different stem cells. Additionally, miR-204 and miR-211 have the same seed-region sequence and, therefore, have theoretically the same targets. Although miR-211 is expressed at a very low level compared with miR-204 in most tissues, it is expressed at a higher level than miR-204 in melanocytes, suggesting a more dominant function in melanogenesis [3]. A high level of miR-211 can interfere with the significance of downregulation of miR-204 in progression of cancer. In future studies, it would be of interest to distinguish the different expression patterns and the functional activities of miR-204/211.

## Abbreviation

AR: Androgen receptor
ccRCC: Clear cell renal cell carcinoma
CRPC: Castration-resistant prostate cancer
CSCs: Cancer stem cells
EEC: Endometrioid endometrial cancer
EMT: Epithelial to mesenchymal transition
VHL: Hippel-Lindau tumor-suppressor gene
MSCs: Mesenchymal stem cells
miRNAs: MicroRNAs
NE: Neuroendocrine
NEPC: Neuroendocrine-like prostate cancer
NSCLC: Non-small cell lung cancer
PDEF: Prostate-derived Ets factor
RPE: Retinal pigment epithelium
PAC: Prostatic adenocarcinoma
SCNC: Small-cell neuroendocrine carcinoma
SSCs: Spermatogonial stem cells
TRPM3: Transient receptor potential melastatin 3 gene
TGF-β: Transforming growth factor β
